# A Case of Septic Encephalopathy Diagnosed Following Non-inflammatory Febrile Response in an Elderly Patient After Sepsis

**DOI:** 10.7759/cureus.46600

**Published:** 2023-10-06

**Authors:** Yuki Amano, Hirotaka Ikeda, Chiaki Sano, Ryuichi Ohta

**Affiliations:** 1 Family Medicine, Shimane University Medical School, Izumo, JPN; 2 Community Care, Unnan City Hospital, Unnan, JPN; 3 Community Medicine Management, Shimane University Faculty of Medicine, Izumo, JPN; 4 Communiy Care, Unnan City Hospital, Unnan, JPN

**Keywords:** elderly patients, post-septic encephalopathy, systemic inflammation, cerebrospinal fluid punctures, autoimmune responses, post-sepsis encephalitis, vascular lesions, sepsis, prolonged consciousness disturbance, intracranial disorders

## Abstract

Intracranial disorders are common in cases of prolonged disturbances of consciousness following sepsis. Among these, investigation of vascular lesions is warranted because only a few patients have encephalitic symptoms. However, the examination may not be comprehensive owing to the lack of rapid changes in the clinical status. This report presents the case of an elderly woman with severe sepsis who experienced prolonged disturbances in consciousness and persistent fever. Lumbar puncture results suggested the possibility of post-sepsis encephalitis. Sepsis induces systemic acute inflammation and activates autoimmune responses, leading to prolonged brain inflammation in some cases. When disturbances in consciousness persist after sepsis, a thorough investigation for the possibility of post-septic encephalitis is imperative.

## Introduction

Unconsciousness often persists after sepsis and has several potential causes [1]. In some cases, sepsis can lead to prolonged disturbances of a patient's sensorium [2]. Furthermore, it is essential to consider the possibility of cerebrovascular diseases resulting from the systemic vascular fragility caused by sepsis. One potential cause is the spread of systemic inflammation caused by sepsis to the brain, triggering autoinflammation [3]. Intracranial inflammation is often not detected using serological tests, leading to delayed diagnosis [4]. In this study, we performed a cerebrospinal fluid puncture on a patient with prolonged unconsciousness and fever after severe sepsis. Following the detection of elevated cerebrospinal fluid protein levels, we treated the patient for post-septic encephalopathy and observed fever reduction and improved consciousness. Herein, we present a case of post-sepsis unconsciousness. Through this case report, we discuss the realistic management of post-sepsis unconsciousness in older patients as society ages, including the possibility of post-septic encephalopathy.

## Case presentation

A 71-year-old woman was transferred to a rural community hospital for rehabilitation following sepsis. Before the transfer, the patient was hospitalized at a university hospital for status epilepticus and septic shock. Blood cultures were positive for *Streptococcus mitis*, which improved after intravenous vancomycin treatment for two weeks. However, her consciousness deteriorated, persistent epileptic activity was observed, and a tracheostomy was performed. Subsequently, the patient was weaned off the ventilator, her consciousness improved, and she was transferred to our hospital for rehabilitation. Before hospitalization, the patient lived with her son and was independent in eating, dressing, and excretion. Her medical history included Parkinson’s disease, left vertebral artery dissection, cholecystectomy for gallstones, uterine fibroids, gastric ulcers, gastroesophageal reflux disease, and psychosomatic disorders. Her medications included levodopa at 300 mg/day and ursodeoxycholic acid at 50 mg/day.

On the day of admission, the patient’s vital signs were as follows: consciousness level, Glasgow coma scale of E4VTM6; temperature, 36.9°C; pulse rate, 63 bpm; blood pressure, 145/84 mmHg; respiration rate, 15 breaths/min; and SpO_2_, 100% (room air). Blood tests showed elevated total bilirubin levels at 2.1 mg/dL, alkaline phosphatase of 384 U/L, and γ-GTP level of 654 IU/L (Table 1).

**Table 1:** Initial laboratory data of the patient CK, creatine kinase; CRP, C-reactive protein; eGFR, estimated glomerular filtration rate; SARS-CoV-2, severe acute respiratory syndrome coronavirus 2; TSH, thyroid-stimulating hormone.

Parameter	Level	Reference
Blood		
White blood cells (×10^3^/μL)	5.6	3.5-9.1
Neutrophils (%)	79.4	44.0-72.0
Lymphocytes (%)	10.0	18.0-59.0
Monocytes (%)	7.7	0.0-12.0
Eosinophils (%)	2.2	0.0-10.0
Basophils (%)	0.7	0.0-3.0
Red blood cells (×10^6^/μL)	3.16	3.76-5.50
Hemoglobin (g/dL)	11.4	11.3-15.2
Hematocrit (%)	32.3	33.4-44.9
Mean corpuscular volume (fL)	101.4	79.0-100.0
Platelets (×10^4^/μL)	25.1	13.0-36.9
Total protein (g/dL)	6.0	6.5-8.3
Albumin (g/dL)	2.8	3.8-5.3
Total bilirubin (mg/dL)	2.1	0.2-1.2
Aspartate aminotransferase (IU/L)	30	8-38
Alanine aminotransferase (IU/L)	8	4-43
Alkaline phosphatase (U/L)	384	106-322
γ-Glutamyl transpeptidase (IU/L)	654	<48
Lactate dehydrogenase (mg/dL)	174	121-245
Blood urea nitrogen (mg/dL)	17.1	8-20
Creatinine (mg/dL)	0.27	0.40-1.10
eGFR (mL/min/1.73 m^2^)	90	>60.0
Serum Na (mEq/L)	138	135-150
Serum K (mEq/L)	3.4	3.5-5.3
Serum Cl (mEq/L)	102	98-110
Serum Ca (mg/dL)	9.2	8.8-10.2
Serum P (mg/dL)	3.6	2.7-4.6
Serum Mg (mg/dL)	1.8	1.8-2.3
CK (U/L)	45	56-244
CRP (mg/dL)	0.08	<0.30
TSH (μIU/mL)	1.08	0.35-4.94
Free T4 (ng/dL)	1.0	0.70-1.48
SARS-CoV-2 antigen	Negative	Negative
Urine		
Leukocyte	Negative	Negative
Nitrite	Negative	Negative
Protein	Negative	Negative
Glucose	Negative	Negative
Urobilinogen	Normal	Normal
Bilirubin	Negative	Negative
Ketones	Negative	Negative
Blood	Negative	Negative
pH	7.0	
Specific gravity	1.016	

**Figure 1:**
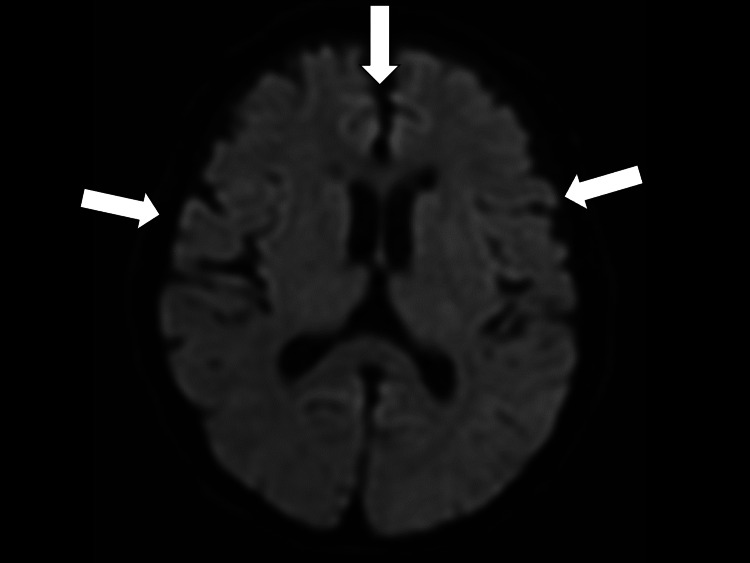
Magnetic resonance imaging (diffusion) showing a high pericerebellar signal (white arrows)

On the fifth day of admission, she experienced recurrent episodes of fever (maximum of 39°C). Blood culture results were negative. Sputum and urine culture tests were conducted to determine the source of the fever, although the results were negative. Given the possibility of sepsis, ampicillin and sulbactam (6 g/day) were administered. On the 11th day of illness, a sputum culture detected highly resistant *Pseudomonas aeruginosa*; thus, the antibiotics were changed to tazobactam/piperacillin. On day 15, magnetic resonance imaging (MRI) of the head was conducted to determine the cause of the persistent fever, which showed a high pericerebellar signal on diffusion MRI (Figure 1).

Magnetic resonance cholangiopancreatography was performed because of elevated biliary enzyme levels; however, no significant findings were noted. A lumbar puncture was performed to identify intracranial lesions. On the same day, the cerebrospinal fluid analysis showed a significantly elevated protein level of 263 mg/dL with a cell count of 1 cell/high-power field and no growth of organisms, prompting the consideration of encephalitis and initiation of methylprednisolone 1000 mg/day for three days, followed by oral prednisolone (40 mg/day). Her consciousness gradually improved, and the temperature decreased to normal levels. On day 18, follow-up lumbar puncture results showed a reduction in protein levels to 205 mg/dL. Oral intake was difficult because of dysphagia; therefore, gastrostomy was performed. Prednisolone was scheduled to be reduced to 10 mg every two weeks. On day 27 of admission, the patient was discharged to a nursing home.

## Discussion

This report presents a case of post-septic encephalopathy in which a prolonged inflammatory response was observed despite appropriate treatments in fever evaluation, such as treatments for pneumonia. Sepsis can trigger various inflammatory responses in the human body, some of which may extend into the cranial cavity and activate autoinflammation. This case suggests that post-septic encephalopathy should be considered a possible cause of prolonged impaired consciousness after sepsis. Even when the inflammatory response is negative, intracranial lesions should be considered, and careful examination and treatment should be provided.

The pathogenesis of post-septic encephalopathy may involve the spread of inflammation into the cranial cavity owing to severe systemic symptoms accompanied by cerebral circulation disorders and the potential for autoimmune activation [5]. Sepsis-associated encephalopathy (SAE) is a diffuse brain dysfunction resulting from a systemic inflammatory response to infection [6,7]. Although the pathophysiology of septic encephalopathy is not fully understood, several factors are believed to play a role. One such factor is the failure of cerebral circulation regulation [5,8]. Among the brain’s regulatory mechanisms, chemical regulation responds to CO_2_ concentrations in the cerebral blood vessels. Low CO_2_ causes cerebral vasoconstriction, whereas high CO_2_ dilates cerebral blood vessels, inhibiting an increase in brain tissue PCO_2_ [8]. With increased CO_2_, blood vessels dilate because of factors secreted from the endothelium, such as nitric oxide (NO), which increases cerebral blood flow. Another regulatory function of cerebral circulation is cerebral autoregulation. In the cerebral vessels, cerebral blood flow is maintained constant within a blood pressure range of 50-150 mmHg. The inflammatory response during sepsis impairs cerebral autoregulation, leading to cerebral ischemia [8].

Second, mitochondrial dysfunction was highlighted. The ability of brain cells to produce adenosine triphosphate (ATP) is reduced by mitochondrial dysfunction caused by the inflammatory cytokines, NO, and hypoxia. During neuroinflammation, intracellular metabolic changes, including reactive oxygen species (ROS) production and superoxide dismutase (SOD) activation, may lead to mitochondrial dysfunction, energy deficiency due to reduced ATP production, and cell apoptosis triggered by the release of cytochrome C. Consequently, inflammation may persist in the cranial cavity [9].

Finally, the breakdown of the blood-brain barrier (BBB) contributes. Cytokine storms throughout the body may stimulate cerebral endothelial cells, increase BBB permeability, and allow leukocytes, macrophages, and other mediators to enter the brain, potentially leading to neuroinflammation [10]. Multiple factors are believed to contribute to functional impairment following sepsis. No specific treatment for septic encephalopathy has been established. Differential diagnoses include infectious, metabolic, vascular, tumor, demyelinating, and inflammatory disorders, all requiring careful exclusion.

In this case, encephalitis may have been induced following sepsis when the immune response was overly activated. Furthermore, inflammation in the cranial cavity may have continuously allowed the penetration of inflammatory cells into the cranial space owing to the breakdown of the BBB, possibly triggering inflammation through an autoimmune mechanism. SAE should be considered a differential diagnosis; however, evaluation within a limited timeframe can be challenging. Older patients' SAE progresses gradually, so the signs of the disease can be missed or challenging to detect [9]. Comprehensive evaluations in acute-phase hospitals with restricted admission durations are also challenging for diagnosis, suggesting that community hospitals and general physicians should conduct thorough investigations and provide treatment when managing such cases [11].

## Conclusions

Post-septic encephalopathy arises from vascular and cellular damage within the cranial cavity because of severe inflammation caused by sepsis and may be irreversible. This condition can be mistaken for post-sepsis wastage and sometimes requires careful examination at community hospitals. Primary care physicians in community hospitals should address prolonged fever with negative inflammatory responses, considering the possibility of post-septic encephalopathy.
